# Tgm1-like transglutaminases in tilapia (*Oreochromis mossambicus*)

**DOI:** 10.1371/journal.pone.0177016

**Published:** 2017-05-04

**Authors:** Sandra I. Rodriguez Cruz, Marjorie A. Phillips, Dietmar Kültz, Robert H. Rice

**Affiliations:** 1 Forensic Science Program, University of California, Davis, California, United States of America; 2 Department of Environmental Toxicology, University of California, Davis, California, United States of America; 3 Department of Animal Science, University of California, Davis, California, United States of America; University of Maryland School of Medicine, UNITED STATES

## Abstract

Among the adaptations of aquatic species during evolution of terrestrial tetrapods was the development of an epidermis preventing desiccation. In present day mammals, keratinocytes of the epidermis, using a membrane-bound transglutaminase (Tgm1), accomplish this function by synthesizing a scaffold of cross-linked protein to which a lipid envelope is attached. This study characterizes the abilities of two homologous transglutaminase isozymes in the teleost fish tilapia to form cross-linked protein structures and their expression in certain tissues. Results indicate they are capable of membrane localization and of generating cellular structures resistant to detergent solubilization. They are both expressed in epithelial cells of the lip, buccal cavity and tips of gill filaments. Adaptation of transglutaminase use in evolution of terrestrial keratinocytes evidently involved refinements in tissue expression, access to suitable substrate proteins and activation of cross-linking during terminal differentiation.

## Introduction

Derived originally from ancestral proteases [1], transglutaminases (Tgms) comprise a superfamily of enzymes that in mammals primarily cross-link proteins. Mammals have some 8 isozymes, plus the catalytically inactive band 4.2 protein found in erythrocyte membranes [2], that carry out numerous functions [3,4]. Among the functions attributed to these enzymes, Tgms 1, 3 and 5 participate in cross-linked envelope formation in mammalian epidermis, Tgm 2 assists in cell adhesion and matrix stabilization, and Tgm 4 assists in reproduction in rodents through semen coagulation. Factor XIIIa acts during clotting to stabilize coagulated fibrin and is essential for maintaining pregnancy [5]. Comparative studies beyond the mammals help extend our knowledge of such functions. To this end, a recent study of transglutaminases in zebrafish has revealed the contribution of a Tgm2-like isozyme to bone mineralization [6], and a recent report of the tissue distribution of Tgm1-like isozymes in medaka (*Oryzias latipes*) raises the possibility of previously unsuspected physiological roles [7].

Studies of transglutaminases in fish may also help elucidate the aquatic to terrestrial transition of a common ancestral species during tetrapod evolution. Among a number of adaptations, development of an epidermis that prevents desiccation was an important permissive factor. The epidermis of terrestrial tetrapods displays abundant keratin proteins with disulfide cross-links that provide structural stability and resistance to friction. Studied most extensively in humans and mice, terminally differentiated epidermal cells of the callus layer at the skin surface also display a lipid envelope that prevents loss of internal water through transpiration. The lipids are covalently attached to a scaffold of protein cross-linked by isopeptide bonds induced by human keratinocyte transglutaminase (TGM1). Genetic defects in TGM1 are a prevalent cause of autosomal recessive congenital ichthyosis, characterized by dry scaly skin and barrier disruption [8,9].

In human keratinocytes, TGM1 is membrane bound by virtue of fatty acid thioesterification [10] near the amino terminus [11] at a cluster of 5 cysteine residues. Transfer of a 32 residue sequence containing this cysteine cluster to the amino terminus of involucrin, a very soluble protein, induces its membrane anchorage [12]. Expressed primarily in keratinocytes, the enzyme produces cross-linked protein structures that resist detergent solubilization after terminal differentiation [13] or after the living cells are permeabilized to permit an increase in cytosolic calcium ion necessary for activation of transglutaminase cross-linking [14]. Detergent treatment reveals empty cell ghosts that can provide a quantitative measure of the effectiveness of substrate protein cross-linking near the plasma membrane [15].

Thanks to genomic sequencing of fish species, the various transglutaminases they express and their functions are now amenable to study. The finding of Tgm1-like isozymes in fish raises questions about their functions in aquatic vertebrates, whether their properties could have been utilized in development of the lipid barrier that appeared in terrestrial tetrapods and whether further adaptations were necessary. To address such questions, the present study concerns structural properties and tissue expression of the two Tgm1-like isozymes present in tilapia *(O*. *mossambicus)*. Facilitated by availability of an epithelial cell line established from the lip of this species [16] that expresses them, both isozymes appear capable of protein cross-linking at the plasma membrane.

## Methods

### Ethics statement

This study was approved by the UC Davis Institutional Animal Care and Use Committee (protocol #13468). Adult tilapia (*O*. *mossambicus*) were kept in dechlorinated freshwater at 25–27°C in a 114 liter recirculating tank on a 12 hr:12 hr (light:dark) photoperiod. Fish were fed daily with a commercial trout pellet diet (Nelson’s Sterling Silver Cup, Murray, Utah, USA) at approximately 1% of fish body mass. Tilapia were stunned with a blow to the head and quickly euthanized by spinal transection. Tissues were immediately collected in 10% buffered formalin and snap-frozen in liquid nitrogen for processing.

### Cell culture

Tilapia lip epithelial cells (OmL) were propagated in DMEM/F12 (2:1) medium supplemented with fetal bovine serum (5%), hydrocortisone (0.4 μg/ml), adenine (0.18 mM), insulin (5 μg/ml) and transferrin (5 μg/ml) supported by a feeder layer of lethally irradiated 3T3 cells as previously described [16]. Epidermal growth factor (10 ng/ml) and Y27632 rho kinase inhibitor (10 μM) from Chemdea (Ridgewood, NJ) were added at 3 to 4 day intervals when the medium was changed. The cells were maintained at 26°C in a 5% carbon dioxide atmosphere. For study of TGM membrane localization, confluent cultures were scraped into 0.4 ml of 20 mM Tris-Cl– 2 mM EDTA containing HALT protease inhibitor cocktail (ThermoFisher Scientific) and stored frozen. Upon thawing, samples were sonicated, examined by light microscopy to verify total cell disruption and separated into soluble and particulate fractions by centrifugation (5 min at 10,000 x g). Resuspending the insoluble material in the pellets, resonication and recentrifugation gave the same result.

### Monoclonal antibodies

Hybridomas were raised by Abmart (Shanghai, China) using SEAL™ library design against the deduced amino acid sequences of two Tgm1-like transglutaminases. After purification of monoclonal antibodies from ascites with an Nab A/G Spin kit (ThermoFisher Scientific), hybridomas were selected by immunoblotting extracts of HEK293FT cells transfected with the full length coding region for the corresponding Tgm1. Selected hybridoma ascites recognizing the epitopes EPSKEDGGNGEK (Tgm1A) and ITDKVEFTKL (Tgm1B) were used as sources of primary antibodies for immunohistochemistry. These peptide epitopes for each Tgm1 were selected in part for their absence in the other Tgm1 sequence, and no cross-reaction between Tgm1A and Tgm1B was observed in immunoblotting. They did not recognize epitopes in HEK293FT cells. Hybridomas were grown at 37°C in RPM1-1640 medium supplemented with 17% fetal bovine serum, penicillin and streptomycin. Culture supernatants were concentrated 10-fold by ammonium sulfate precipitation (0.28 g/ml), dialyzed against 0.2 M NaCl– 0.05 M Tris-Cl buffer (pH 8) and used for immunoblotting.

### Real time PCR

Custom Taqman assays for tilapia mRNAs for the two transglutaminases and 3 housekeeping genes (Talin1, Filamin A, S2-40S) were purchased from Applied Biosystems/Life Technologies (Grand Island, NY). Primer sequences, derived from the mRNA sequence of the closely related *O*. *niloticus*, are given in S1 Table. Normalization using the δδCt method [17] based on the geometric mean of Ct values for 3 housekeeping genes (Talin1, Filamin A, S240S) gave essentially the same relative amounts of Tgm1A and Tgm1B.

### Transfection

RNA was prepared from confluent tilapia lip OmL cultures using Trizol (ThermoFisher). cDNA was synthesized from it using a New England BioLabs (Beverly, MA) AMV First Strand cDNA Synthesis kit and, with primers given in S2 Table, used as a template for PCR amplification of the full length coding regions of Tgm1A and Tgm1B. The PCR products were cloned into pCDNA3.1 using a Gibson Assembly kit from New England BioLabs. Constructs were verified by sequencing of the 5’ and 3’ ends. A 3X FLAG epitope, encoding the peptide sequence DYKDHDGDYKDHDDYKDDDDK, was added at the 3’ end of the Tgm1 sequences by cloning double stranded oligonucleotides with appropriate overhangs into the XhoI and Asp718 sites of the cDNA constructs. Constructs with deletion of the 5 amino acids in the cysteine cluster (CPCCC) or their replacement by AIAAA (with an MscI restriction site introduced for screening) were prepared using the New England BioLabs Q5 Site-Directed Mutagenesis Kit (primers given in S3 Table). HEK293FT cells (Invitrogen) were transfected with the cDNAs using Lipofectamine 2000 (Thermo-Fisher).

### Immunoblotting

Protein extracts for hybridoma screening were prepared by dissolving transfected HEK293FT cells in 10 mM Tris (pH 7.5) containing 2% SDS. After protein quantitation by BCA assay (Pierce), dithiothreitol (DTT) was added to 20 mM, and extracts were incubated for at least 15 minutes at 37°C. 20 μg of protein were separated by SDS polyacrylamide gel electrophoresis and transferred to Immobilon-P membranes (Millipore). Membranes were blocked in 25 mM Tris (pH 7.5), 150 mM NaCl, 0.05% Tween 20 (TBST) containing 5% nonfat dry milk. Membranes were incubated overnight at 4°C with primary antibody diluted in blocking buffer, washed in TBST buffer, then incubated for one hour with horseradish peroxidase conjugated anti-mouse secondary antibody (Cell Signaling Technology) diluted in blocking buffer, followed by several washes in TBST buffer. Bands were visualized with ECL2 reagents (Pierce) using a Thermo Pierce MyECL imager.

### Cross-linked envelope quantitation

Confluent OmL cultures were treated overnight with or without X537A at a concentration of 50 μg/ml in serum free medium, then adjusted to 2% in SDS and 20 mM in DTT. The ionophore permits rapid calcium entry, activating transglutaminase cross-linking, and is lethal to the cells. Since the detergent treatment dissolves cellular proteins that are not isopeptide cross-linked, cells not treated with ionophore dissolve (S1 Fig). Detergent treatment permits the envelopes in ionophore-treated cells to be seen by light microscopy and to be isolated. After 1 hr of treatment with SDS and DTT, the DNA from lysed cells was fragmented by passage through a 22 gauge needle, reducing the viscosity of the solution, and the envelopes were recovered by low speed centrifugation, rinsed in 0.1% SDS and resuspended in 1 ml of 0.1% SDS. Relative envelope amounts were measured by light scattering (A^340^) as previously described for human epidermal cells [14,15]. More accurate quantitation using disaggregated cells was not feasible, since trypsinization greatly attenuated envelope yield.

### Immunohistochemical staining

Tissues were fixed in 10% buffered formalin, dehydrated in graded alcohol, cleared in xylene and infiltrated with paraffin. Sections (5 μm) were collected on poly-L-lysine coated glass slides (Sigma-Aldrich, St. Louis, MO), air dried at 37°C overnight, deparaffinized in xylene and rehydrated in graded ethanol and then deionized water. Sections were submerged in 10 mM Tris-Cl, 1 mM EDTA, 0.05% Tween 20 (pH 9.0 to 9.2) and microwaved for 3–5 min to retrieve epitopes masked during tissue fixation [18,19]. Sections were then incubated for 30 min in hydrogen peroxide (2.5% in methanol) to eliminate endogenous peroxidase activity, followed by incubation in 100% ethanol for 1 min, hydration in deionized water, rinsing with 0.01% Triton-phosphate-buffered saline (TPBS) and then with phosphate buffered saline. Sections were blocked with 10% goat serum for 30 min at room temperature and then incubated overnight at 4°C with the primary antibody diluted 10 fold with 1% bovine serum albumin in PBS. The slides were then washed once with TPBS, four times with PBS and incubated for 30 min with biotin conjugated goat anti-mouse antibody (1:500 in 1% BSA/PBS) (Jackson ImmunoResearch Laboratories, Inc., West Grove, PA). The slides were washed once with TPBS, four times with PBS, and incubated for 30 min with diluted horseradish peroxidase-conjugated streptavidin (1:1000 in 1% BSA/PBS) (Jackson ImmunoResearch Laboratories, Inc). The slides were rinsed once with TPBS, four times with PBS and immersed in 0.05% 3,3’-diaminobenzidine (Bio Rad Laboratories, Hercules, CA) in 0.1% TPBS buffer containing 0.0015% hydrogen peroxide for 5 min and rinsed with tap water to stop the reaction. Then the sections were counterstained with 10% Gill’s hematoxylin II for 1 min (American Master Tech Scientific, Inc, Lodi, CA), rinsed in deionized water, dipped twice in acid alcohol, rinsed in deionized water, immersed in lithium carbonate until a blue cast was visible, dehydrated once in 95% ethanol and twice in 100% ethanol, each for 5 min, cleared in 50:50 ethanol/xylene and twice in xylene for 5 min and mounted using ClearMount (American Master Tech Scientific, Inc). Negative controls were performed by replacing the primary antibody with 1% BSA diluted in phosphate buffered saline. Immunostained tissue sections were examined by light microscopy and photographed (Olympus BH2-RFCA with Leica DFC 500 camera).

#### Periodic acid-Schiff staining

Adapted from Carson and Hladik Cappellano (2015) [20], the sections were air dried, deparaffinized and rehydrated as above, immersed in 3% acetic acid for 3 min, rinsed in water, treated for 10 min in 0.5% periodic acid, rinsed in water and incubated 15 min in Schiff reagent. After rinsing in water for 5 min for color development, slides were counterstained with hematoxylin (Gill III) for 30 sec (Poly Sciences, Inc, Warrington, PA), then immersed in 80% alcohol (15 dips) and Eosin Y for 1–2 min, dehydrated alcohol, then xylene and mounted using cytoseal 60 (Richard-Allan Scientific) as above. Negative controls omitted the Schiff reagent treatment.

## Results

**Fig 1:**
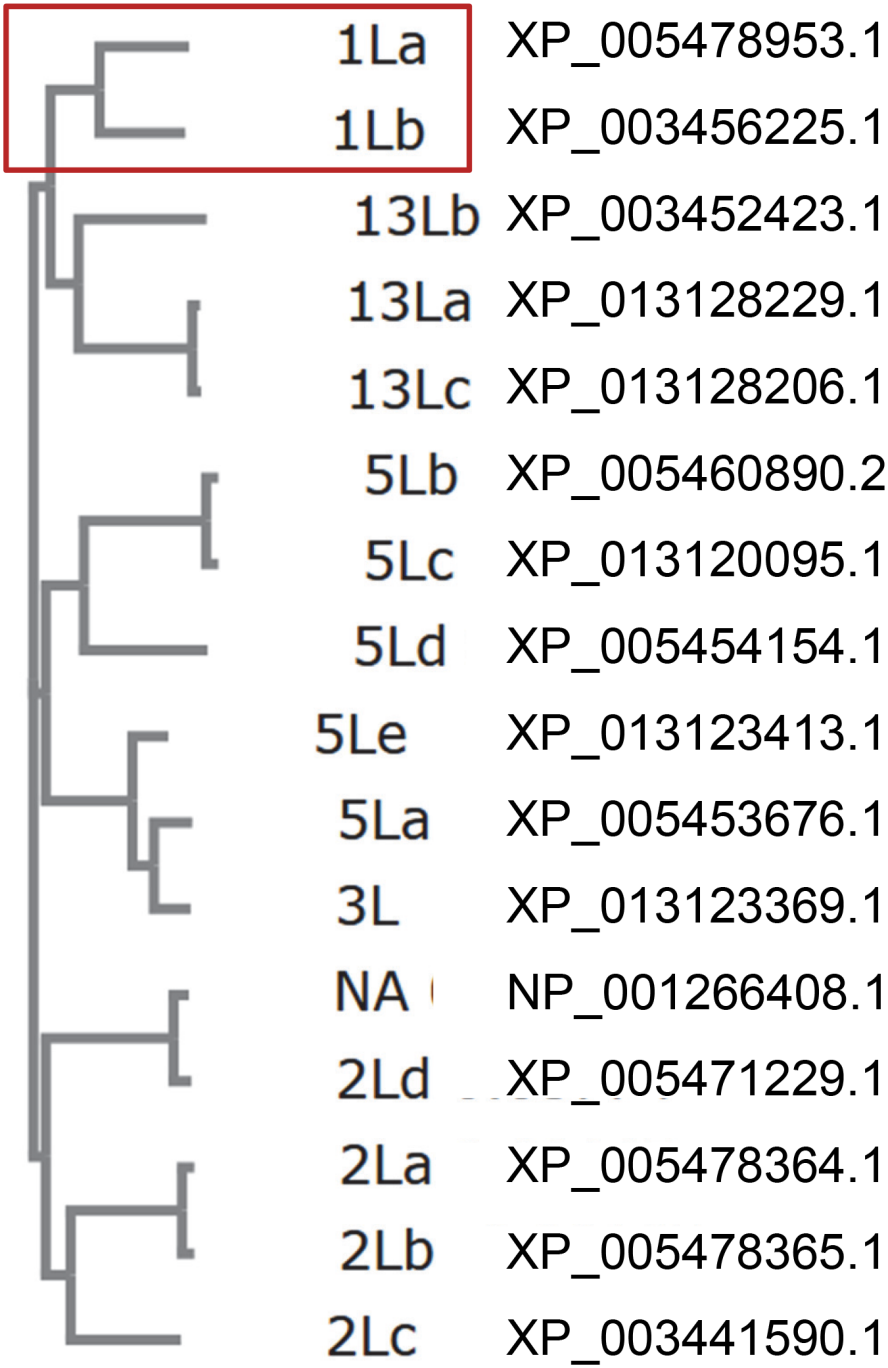
Cladogram of *O*. *niloticus* transglutaminase sequences. Genbank was searched using BLASTP for the active site motif “GQCWVF”. Sequences are identified in the left margin by number: 1, 2, 3, 5, 13 (XIIIa) and NA (not annotated); L, like; lower case letters arbitrarily distinguish those of the same type. The sequences identified as transglutaminases were aligned using the Clustal Omega algorithm with default parameters (available at http://www.ebi.ac.uk/Tools/services/web/toolresult.ebi?jobId=clustalo-I20160718-224602-0849-78264976-oy&analysis=phylotree) to generate the cladogram showing relative estimated evolutionary distances. A red box shows the two Tgm1-like (1La and 1Lb) sequences. The sequence with an inactivating cysteine to serine substitution in the active site (Genbank accession XP_005461247.1) was not included. Entries 5Lb and 5Lc differ by the former having a single extra amino acid, and thus they may not be distinct. Genbank accession numbers are given next to the Tgm.

Interrogation of the NCBI protein database from *O*. *niloticus* using BLASTP with the highly conserved active site motif “GQCWVF” revealed 16 sequences in the transglutaminase family of enzymes (S2 Fig). A cladogram based on multiple sequence alignment using Clustal Omega (Fig 1) contained 2 distinct entries classified as Tgm1-like, 4 as Tgm2-like, 1 as Tgm3-like, 5 as Tgm5-like, 3 as Factor XIIIa-like, and 1 was unclassified but most closely resembled Tgm2. (A sequence annotated as Tgm2-like, lacking much of the N-terminus present in the others, was not included because the active site cysteine was replaced by serine, preventing enzymatic activity.) This finding resembles the tally of 13 Tgms reported for zebrafish [6], which is about average for the 12 species listed in Table 1 (12.6 ± 5) ascertained by searching the NCBI database.

**Table 1:** Cysteine clusters in amino terminal segments of Tgm1s in teleosts and tetrapods.

Species	Aligned sequence^a^	Category^b^	Accession #
*Oreochromis niloticus* (Tilapia)	A**C**QEWLRKV**C**P**CCC**PKH G**C**LWWLRKM**C**P**CCC**KHP	Perciformes^c^	XP_019206699.1 XP_003456225.1
*Takifugu rubripes* (Fugu)	A**C**RQWLRKI**C**P**CC**HPKP G**C**RRWLRKAFP**CCC**QRQ	Tetraodontiformes	XP_011612863.1 XP_003975933.2
*Oryzias latipes* (Medaka)	G**C**QAWWRRV**C**P**CCC**PQV G**C**RRWFRLI**C**P**CCC**KRQ	Beloniformes	XP_004067653.1 XP_011484869.1
*Clupea harengus* (Atlantic herring)	G**C**HRWLRRI**C**P**CCC**RQK A**C**RRWFRKV**C**P**CCC**RTP	Clupeiformes	XP_012690693.1 XP_012690839.1
*Xiphophorus maculatus* (Platyfish)	A**C**RRWLRKA**C**P**CC**YKRP G**C**RRWFRKI**C**P**CCC**KRQ	Cyprinodontiformes	XP_014329032.1 XP_005811105.1
*Salmo salar* (Atlantic salmon)	V**C**RQWLRKI**C**S**CCC**QRL G**C**RQWLRKI**C**P**CCC**QRP S**C**RRWFQKM**C**P**CCC**RRQ G**C**LQ**C**FQKI-F**CC**SPRH	Salmoniformes^c^	XP_014072265.1 XP_014024843.1 NP_001133569.1 XP_014047013.1
*Danio rerio* (Zebrafish)	S**C**GAWFRQ**CC-C**S**C**RKS G**C**RRWLRKA**C**P**CC**LRRQ	Cypriniformes^c^	XP_009294851.1 XP_694950.3
*Cynoglossus semilaevis* (Tongue sole)	P**C**KLWLRKV**C**P**CC**RPKP S**C**RRWFRKM**C**P**CCC**KRQ	Pleuronectiformes	XP_008305375.1 XP_008332432.1
*Ictalurus punctatus* (Channel catfish)	G**C**RRWFRKV**C**P**CCC**RRQ K**C**RRWFRKI**C**P**CCC**RQA	Siluriformes^c^	XP_017351706.1 XP_017341995.1^d^
*Esox Lucius* (Northern pike)	A**C**RRWWRKM**C**P**CCC**KKR	Esociformes	XP_010864004.1
*Scleropages formosus* (Asian Arowana)	G**C**WRWLQKM**C**P**CCC**RKQ	Osteoglossiformes	KPP72492.1
*Astyanax mexicanus* (Mexican tetra)	P**C**KSWFRKL**C**P**CCC**QQD	Characiformes^c^	XP_007233483.1
*Xenopus tropicalis*	RKKSFWERL**C**P**CCC**TER	Amphibian	XP_002939073.1
*Alligator mississippiensis*	RRRGVFSKV**C**A**CC**R**CC**A	Reptile	XP_006259796.2
*Monodelphis domestica*	SGRSFWAR**CC**S**CC**S**C**RG	Marsupial	XP_001380226.1
*Homo sapiens*	GGRSFWAR**CC**G**CC**S**C**RN	Mammal (placental)	NP_000350.1

^a^Amino acid sequence near amino terminus taken from the complete Tgm1 sequences aligned using Clustal Omega. Other teleost species that have Tgm1-like sequences with cysteine clusters include *Sinocyclocheilus graham*, *Sinocyclocheilus rhinocerous*, *Sinocyclocheilus anshuiensis*, *Kryptolebias marmoratus*, *Poecilia Mexicana* (Cypriniformes); *Larimichthys crocea*, *Stegastes partitus*, *Maylandia zebra*, *Haplochromis burtoni*, *Neolamprologus brichardi*, *Pundamilia nyererei*, *Notothenia coriiceps* (Perciformes); *Nothobranchius furzeri*, *Cyprinodon variegatus* (Ciprinodontiformes). Among the Aves, *Ficedula albicollis* exhibits a Tgm1 sequence with a cysteine cluster near the amino terminus.

^b^Phylogenetic order of teleosts and class of tetrapods.

^c^Phylogenetic orders in which horny features have been reported. Others are Atheriniformes, Gonorhynchiformes and Scorpaeniformes [24–27].

^d^This sequence appears to be a fusion of two Tgm1-like sequences (with two GQCWVF active site regions), the second of which has the cysteine cluster.

Close inspection of the Tgm1-like protein sequences from *O*. *niloticus* revealed a cysteine cluster (CPCCC) in the amino terminal segment resembling that in mammalian Tgm1s. This feature was present in Tgm1-like sequences generally among the fish species whose genome sequences were available in the NCBI database. Table 1 shows a lineup of the cysteine cluster regions in representative species from 12 phylogenetic orders of fish that were aligned over their entire lengths (S3 Fig). The table also shows the corresponding cysteine cluster regions in representative amphibian, reptile, marsupial and primate sequences. The terrestrial species have a single Tgm1, while the fish commonly have more than one, likely reflecting the teleost-specific genome duplication since the divergence of fish and mammals and, in the case of Atlantic salmon (4 sequences), an additional, more recent salmonid-specific genome duplication [21,22]. Tilapia Tgm1A and Tgm1B exhibit higher degrees of amino acid identity toward each other (63%) than either does toward human TGM1 (49%), consistent with gene duplication after divergence of the fish from the mammalian lineage.

**Fig 2:**
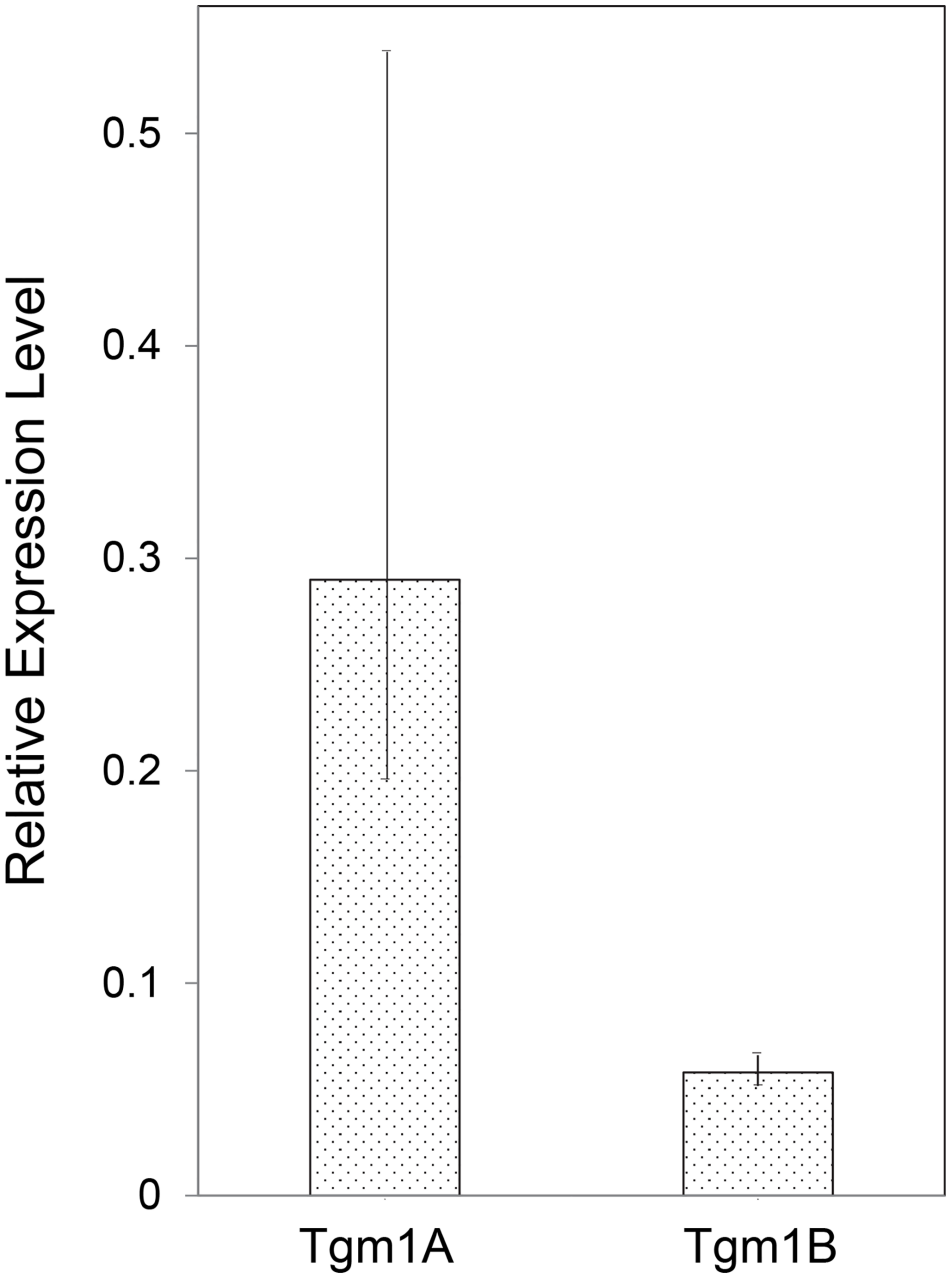
Relative levels of Tgm1A and Tgm1B mRNA in cultured lip cells. Values are presented for 3 independent samples (mean ± std dev) relative to those of the housekeeping gene, Talin1.

The finding that Tgm1-like enzymes containing cysteine clusters near the amino termini are widespread among teleosts suggested that this feature functions as it does in mammals. That fish Tgm1-like enzymes are capable of making cross-linked protein structures resembling those in mammalian keratinocytes was tested using these Tgm1s from *O*. *mossambicus*. Since the genome sequence was not available for this species, cDNA clones of Tgm1 from it were obtained using proteomic and nucleotide database information from *O*. *niloticus*. This option appeared viable since these are sometimes considered subspecies, hybridizing readily to yield fertile offspring. Sequencing a total of 1750 bases (≈850 bp from each end of the PCR products) yielded 99% identity with the *O*. *niloticus* cDNA sequence in Genbank. PCR primers were then designed for measuring the Tgm expression in the OmL epithelial cell line derived from the lip of the fish. Judging by real time PCR, the mRNAs for the Tgm1-like enzymes were both expressed, with Tgm1A being several fold the level of Tgm1B (Fig 2).

**Fig 3:**
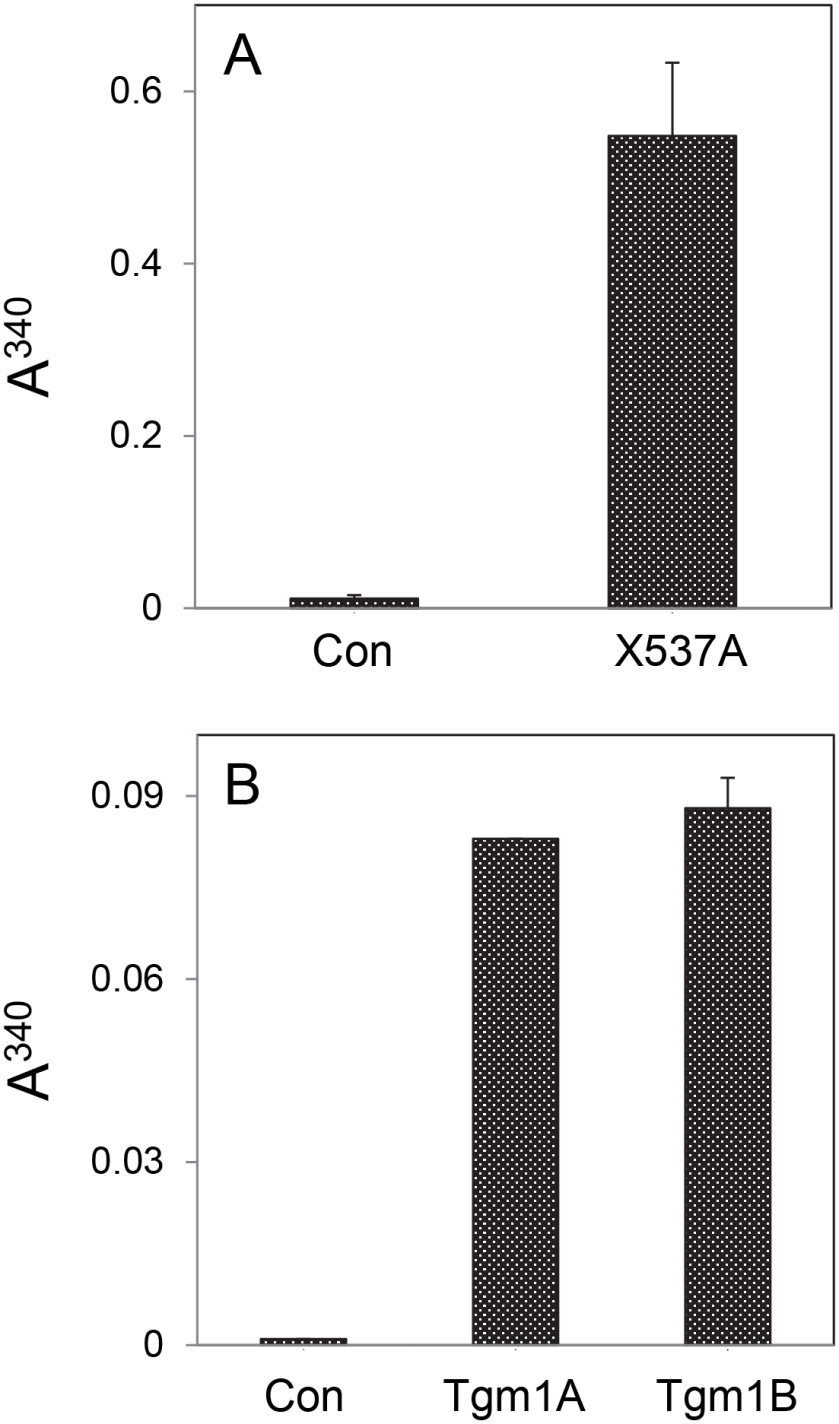
Stimulation of envelope cross-linking by the ionophore X537A. Shown is the degree of light scattering (A^340^) by cross-linked envelopes isolated after addition of SDS and DTT. (A) Confluent cultures of OmL cells with (X537A) and without (Con) overnight treatment with ionophore; compilation of 4 independent experiments. (B) HEK293FT cells not transfected (Con) or transfected with the full length coding region of Tgm1A or Tgm1B; two days after transfection, cultures were treated overnight with ionophore; compilation of two independent experiments. In each panel, differences between treated and control samples were judged significant (p<0.01) by ANOVA using STATA SE9 statistical software.

**Fig 4:**
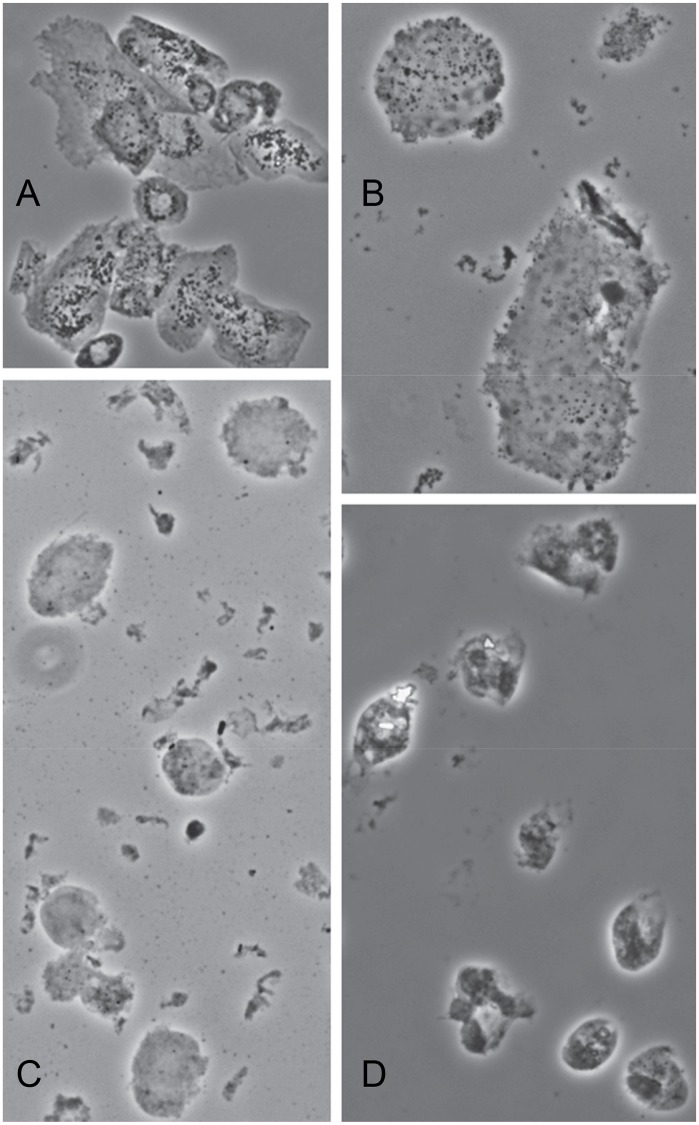
Envelopes from cultured lip and HEK293 cells, all treated with SDS and DTT. Confluent OmL cells were treated overnight with X537A (A) or harvested after spontaneous detachment (B). HEK293FT cells were transfected with Tgm1A (C) or Tgm1B (D) full length coding regions for two days and then overnight with X537A ionophore.

Since tilapia lip OmL epithelial cells expressed Tgm1A and B, they were tested for their ability to synthesize cross-linked envelopes. Adherent cultures, when treated with detergent and reducing agent (2% SDS—20 mM DTT), were nearly completely dissolved. However, treating them overnight with the ionophore X537A, activating translgutaminase activity through calcium influx, and then with detergent and reducing agent prevented the large majority of cells from dissolving, revealing the envelopes they synthesized. As estimated by light scattering of the insoluble material isolated by centrifugation (Fig 3A), detergent-resistant structures increased >50 fold. Microscopically, the cell remnants appeared to be ghosts with interiors that contained much granular material (Fig 4A). When such cultures were held at confluence, several hundred cells were spontaneously released daily that could be harvested at each medium change. Treating those cells with SDS and reducing agent did not dissolve them but produced ghosts with much granular material (Fig 4B) analogous to keratinocyte squames harvested from confluent human epidermal cultures [23]. The latter cease protein synthesis when deprived of attachment to the culture dish and become permeable, thereby activating their TGM1 activity even without ionophore addition [24]. In four experiments, 21 ± 7% of the released tilapia cells were estimated to form envelopes, although some appeared incomplete or fragmentary. Suspending the cells in medium without ionophore for a day after removal from the dish by EDTA treatment gave a similar estimate.

To test whether these transglutaminase activities could account for the cellular resistance to being dissolved by detergent, the full length coding region for each, molecularly cloned, was expressed in HEK293 cells. Cultures transfected with either Tgm1 coding region made many detergent-resistant envelopes (Fig 4C and 4D). This phenomenon occurred when the cells were treated with X537A but not if the ionophore treatment was omitted (Fig 3B). By contrast, the HEK cells, either untransfected or transfected with empty vector, dissolved when treated with ionophore and then with detergent and DTT (S3 Fig).

**Fig 5:**
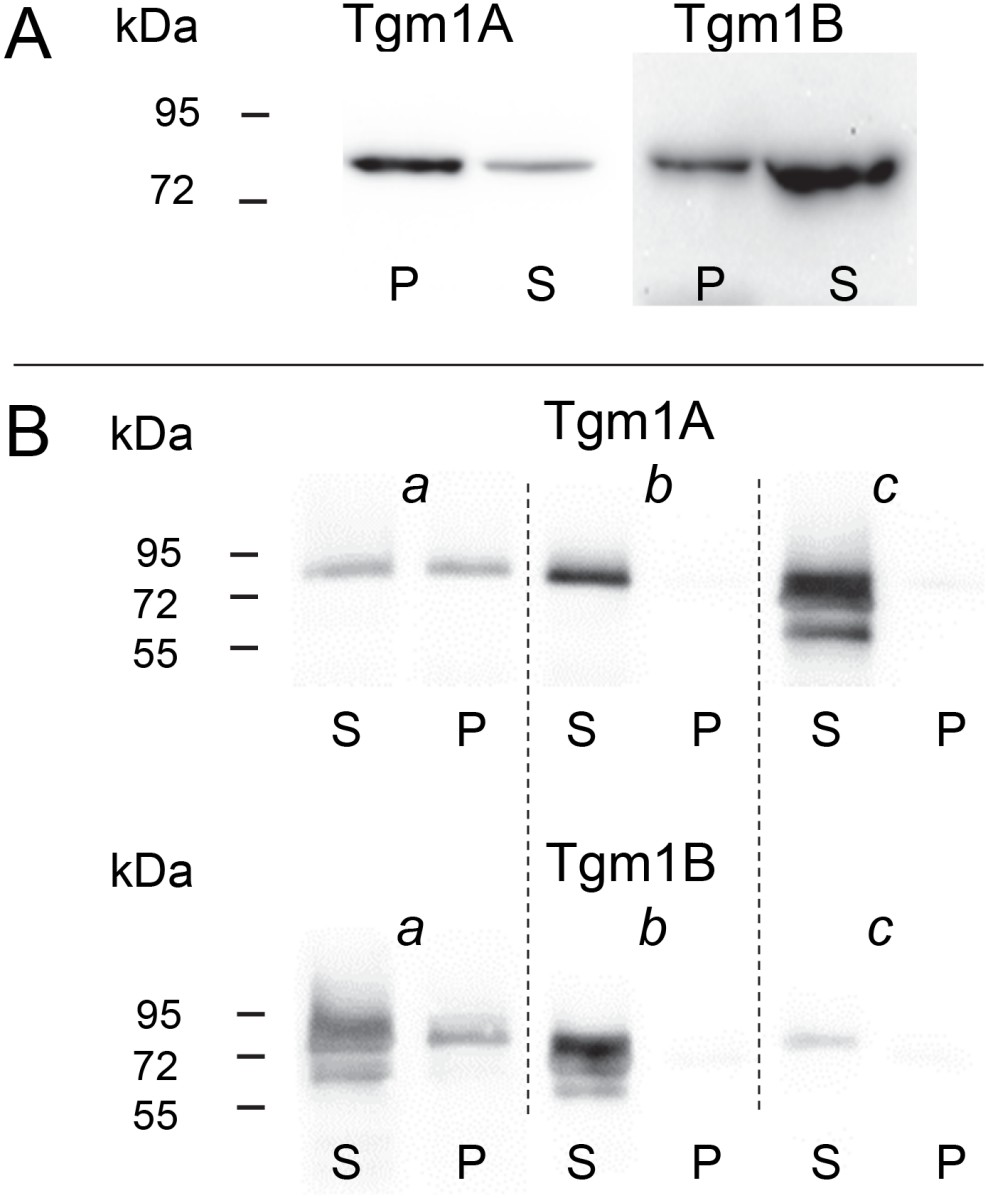
Membrane anchorage of Tgm1A and Tgm1B. (A) Extracts from cultured lip cells showed immunoreactive bands with the expected mobilities of 86 kDa (784 and 786 amino acids, respectively) in both particulate (P) and soluble (S) fractions.(B) Extracts from HEK293 cells also showed substantial immunoreactive bands in particulate and soluble fractions when transfected with cDNA encoding the wild type coding region (a). Transfections with constructs where the cysteine cluster (CPCCC) was deleted (b) or was replaced by AIAAA (c) exhibited little if any Tgm1 in the particulate fraction.

To find whether the two tilapia enzymes were membrane-bound, soluble and particulate fractions of confluent OmL cultures were analyzed by SDS detergent gel electrophoresis (Fig 5A). Substantial Tgm1A and TGM1B were found in the particulate fraction. Confirming the ability of both isozymes to adopt membrane binding properties, when the coding regions were transfected into HEK293 cells, the enzymes were distributed in the soluble and particulate fractions as expressed in OmL cells (Fig 5B). The importance of the cysteine cluster for membrane association was demonstrated by deleting these 5 amino acids (CPCCC) or their mutation to AIAAA. In either case, little if any of the protein was associated with the particulate fraction. A more drastic modification of deleting amino acids 3–94, corresponding to the N-terminal extension in mammals [25], also prevented association with the particulate fraction (not shown).

**Fig 6:**
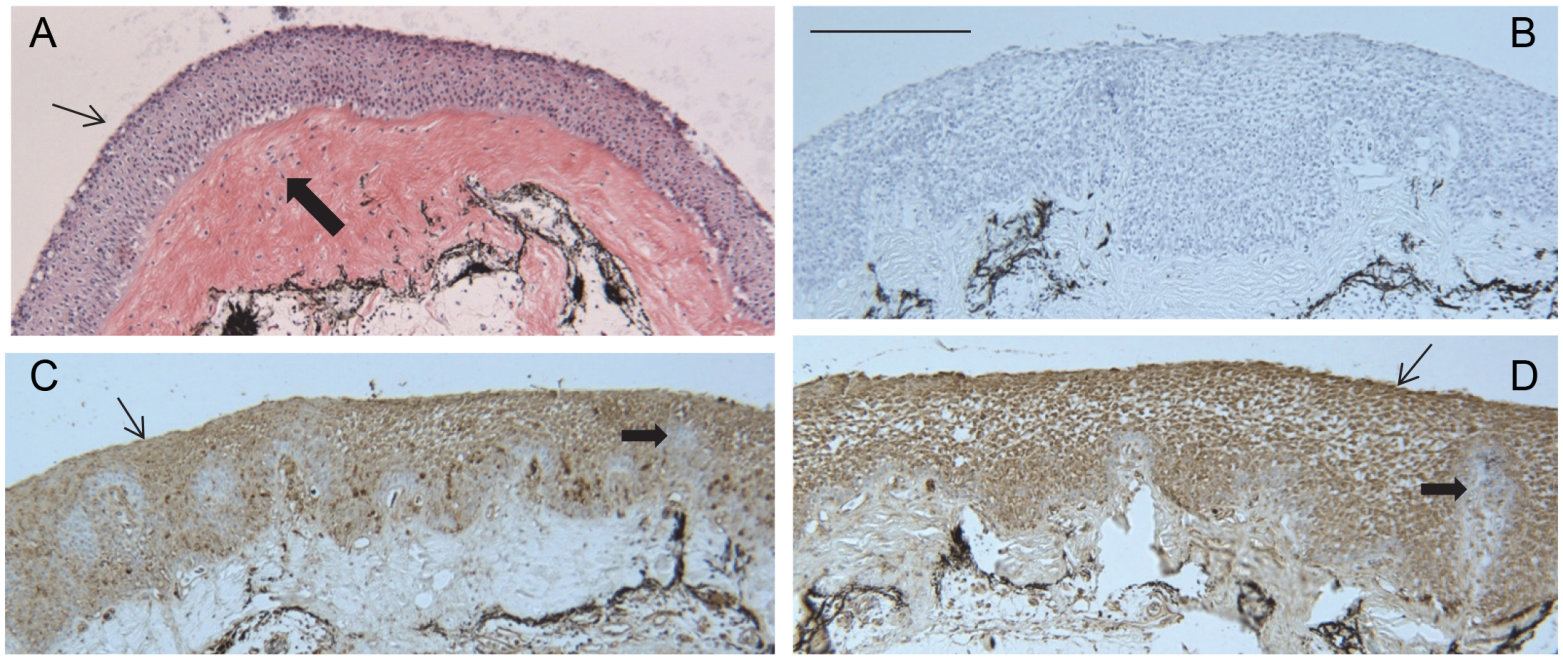
Sections of lip. (A) Hematoxylin and eosin stained. Thin arrow points to epithelium with nucleated cells; thick arrow points to a wide layer of connective tissue with abundant eosin-positive collagen. (B) Immunostained section without primary antibody (negative control). (C) The thin arrow points to Tgm1A immunoreactivity (brown) seen throughout the epithelium, although not uniformly. Some nests of cells, primarily in the lower half of the epithelium and connected to the basal layer, showed lower immunoreactivity than elsewhere (example shown by thick arrow on the right). (D) Tgm1B immunoreactivity (brown) exhibited the same pattern as Tgm1A. The thin arrow points to the brown-stained epithelium, and the thick arrow at right shows a region of lower stain. Each section was counterstained with hematoxylin. Scale bar = 100 μm.

**Fig 7:**
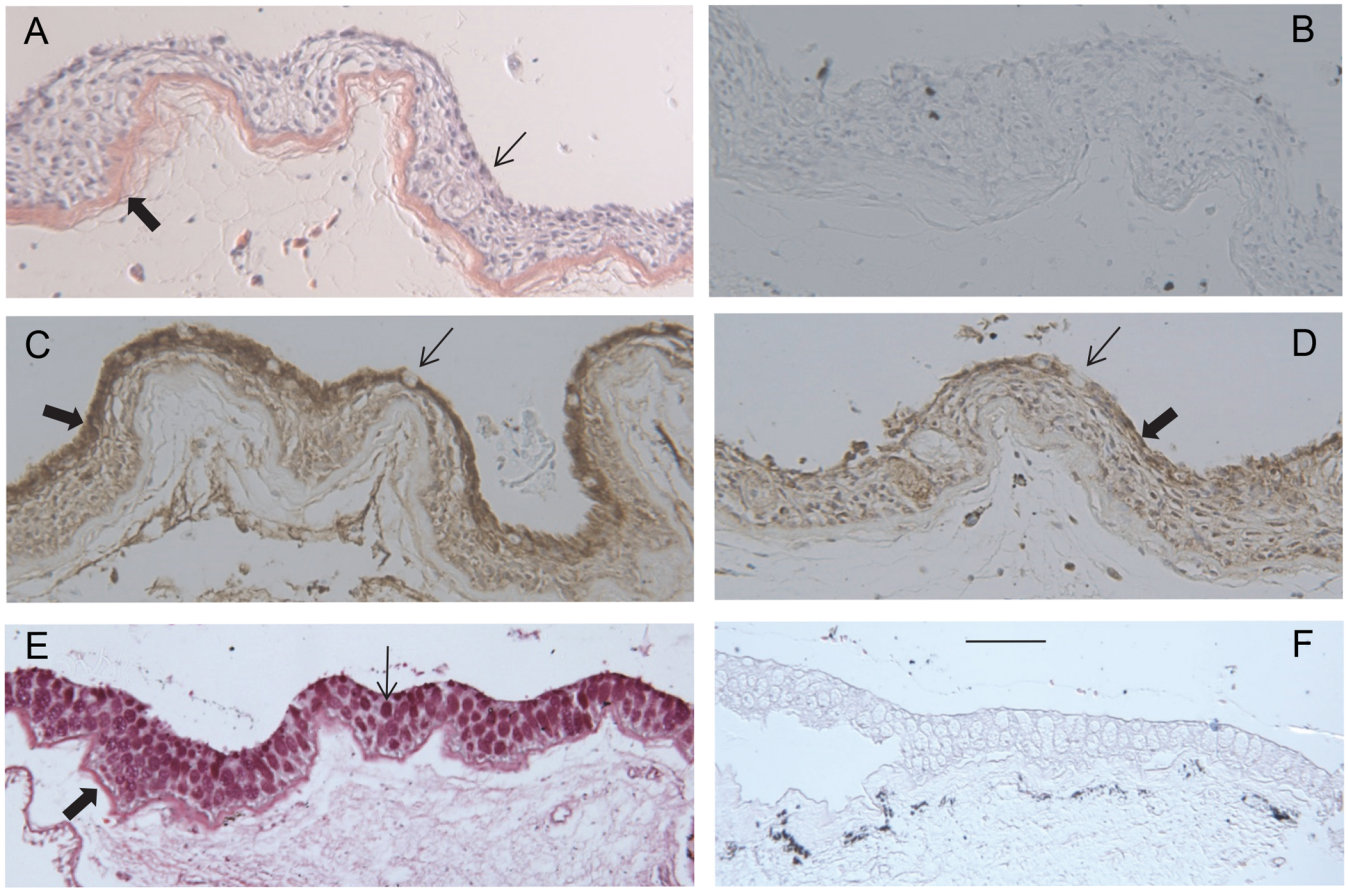
Sections of buccal epithelium. (A) Hematoxylin and eosin stained. Thin arrow points to the stratified epithelium with nucleated cells; thick arrow points to eosin-positive collagen at the border with underlying connective tissue. (B) Immunostained section without primary antibody (negative control). (C) Tgm1A immunoreactivity. The thick arrow points to most concentrated staining of cells at the surface of the epithelium, and the thin arrow points to a glandular cell at the surface releasing mucus. (D) Tgm1B immunoreactivity. Arrows as in C. (E) Periodic acid-Schiff stain showing high density of mucus cells (thin arrow). Thick arrow points to collagen at interface with connective tissue. (F) negative control for section E. Each section was counterstained with hematoxylin. Scale bar = 100 μm.

**Fig 8:**
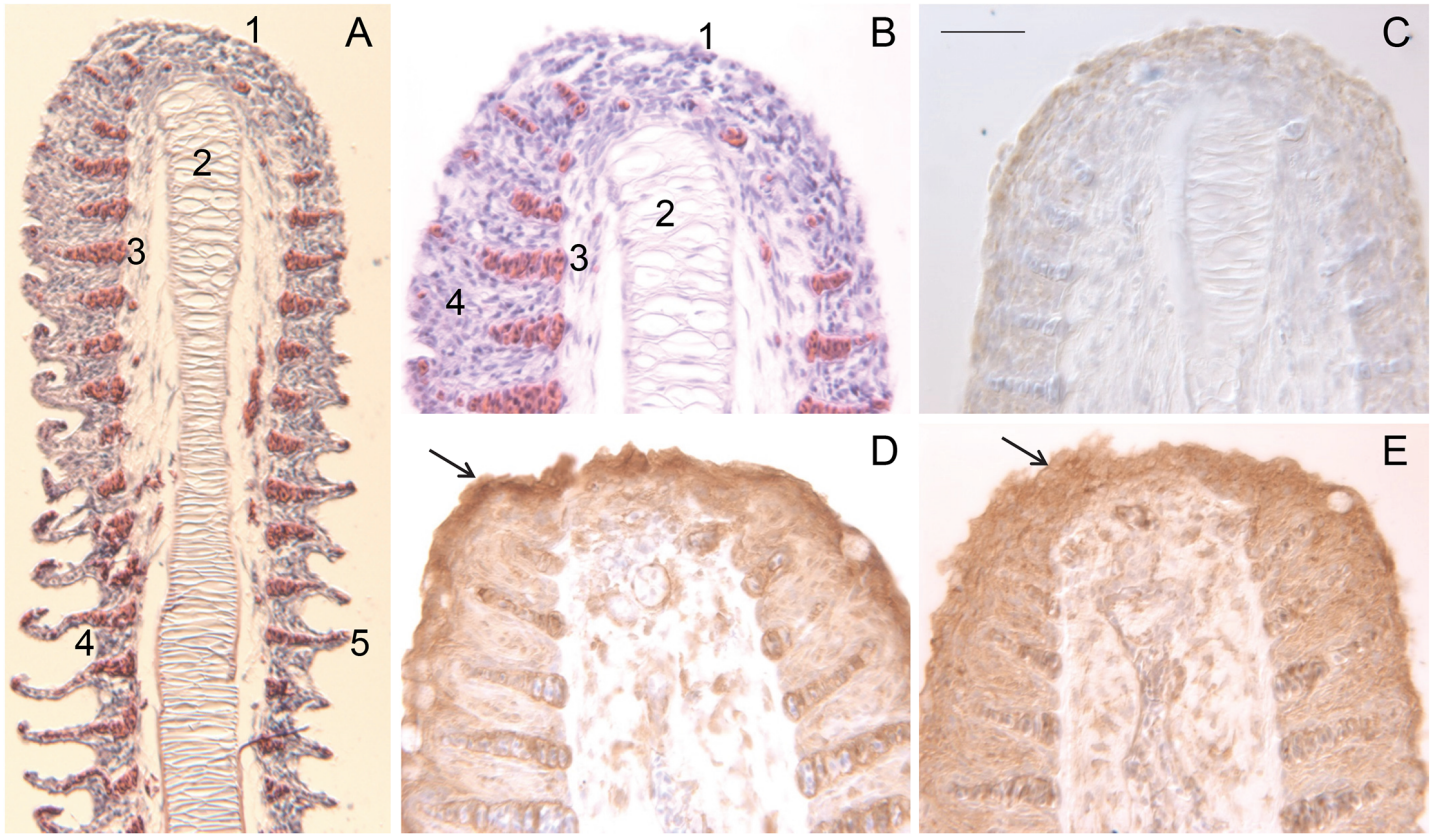
Sections of gill filaments. (A) Low magnification of filament stained with hematoxylin and eosin. Features include the stratified epithelium at the tip of the filament (1), sinus venosus (2), sinus with erythrocytes (3), intralamellar filament epithelium (4) and secondary lamella (5). (B) Hematoxylin and eosin-stained section at higher magnification showing features 1–4 in section A. (C) Immunostained section without primary antibody (negative control); (D) Tgm1A immunoreactivity. Arrow points to most intense staining at the tip. (E) Tgm1B immunoreactivity. Similar to section D, the arrow points to the most intense staining at the tip. Each section was counterstained with hematoxylin. Scale bar = 34 μm (A) and 50 μm (B-E).

To localize the transglutaminase in tissues of adult tilapia, sections of various paraffin-embedded tissues were examined immunohistochemically. The presence of an antigen of ≈90 kDa (similar in mobility to the immunoreactive protein from OmL cells) was confirmed by immunoblotting extracts of lip and buccal tissue (S4 Fig). As seen in Fig 6, lip epithelium is highly stratified and stained strongly for both Tgm1A and Tgm1B in the most superficial cells. Similarly, buccal epithelium was also immunopositive (Fig 7C and 7D). The staining was most intense in the most superficial cells. Certain regions lacked staining, which corresponded to glandular structures visible by periodic acid-Schiff staining (Fig 7E). Cells in the gill were also immunopositive for both isozymes. Staining was strongest at the tips of the stratified epithelium of the filaments and gradually decreased with distance from them (Fig 8C and 8D). By contrast, immunostaining of esophageal epithelium and operculum was low (not shown).

## Discussion

Although teleost fish epidermis ordinarily is not keratinized, some features reminiscent of epidermis of terrestrial tetrapods have been observed. For example, horny structures called “unculi” or “epidermal brushes”, associated with epithelia of the mouths (lips, jaw sheaths) in certain species, appear to be adaptations for foraging [26–28], where epithelial stratification and formation of horny surfaces provide protection against abrasion. Functions proposed for similar horny “breeding tubercles” or “contact organs” observed in the epidermis include protection of body surfaces during contact in spawning and defense or during nest building [29]. Such structures have been reported in at least 8 teleost orders, and Tgm1-like enzymes are present in at least 5 of them as seen by database searching (Table 1). A parallel to mammalian epidermis has been observed in zebrafish epidermis, where a TAp63/p53/Notch/caspase signaling pathway was necessary for stratification of the tubercles, which expressed keratin 8 and Tgm1 in upper layers [30]. In the present case, Tgm1A and B expression was most intense at several sites at epithelial surfaces that might benefit most from resistance to friction.

Evolutionary adaptations often appear to have been selected when pre-existing features were employed in new ways that proved to be advantageous. Present results illustrate that Tgm1-like enzymes in tilapia have properties conducive to development of an epidermis advantageous for a terrestrial environment. Thus, the enzymes have the capability for membrane localization, important for making a protein scaffold at the cell surface for deposition of the lipid envelope. Although not yet investigated, we hypothesize one or more of the numerous protein palmitoyltransferase enzymes encoded in the Tilapia genome recognizes the cysteine cluster near the Tgm1 amino terminus, thereby facilitating its membrane localization, analogous to human TGM1. Like the latter [31], tilapia Tgm1A and Tgm1B are largely hydrophilic proteins that lack membrane spanning segments judging by Kyte-Doolittle hydropathy plotting (not shown).

In addition to membrane-localized Tgm1 activity and possible mechanisms of its activation, cross-linked protein envelope formation may be assisted by other Tgms and also requires substrate proteins that become cross-linked. Lack of known substrates such as involucrin and loricrin in fish could be responsible for the lack of observed envelopes in zebrafish tubercles [30]. Nevertheless, keratinized catfish epidermal cells were reported to form deposits beneath the plasma membrane resembling envelopes, but often these did not extend entirely around the cell margins [32]. Lack of envelopes in zebrafish tubercles could reflect both of these factors as well as an inability to activate the Tgm1 by permitting calcium influx, a key feature occurring in mammalian cornification [33]. Cultured OmL cells were able to make envelopes only when the calcium ion was provided by ionophore treatment or resulting from detachment (desquamation). This likely reflects the phenomenon that, as shown in mouse epidermis, envelopes incorporate many prevalent proteins in the cell, helping to rationalize the mild phenotype of targeted mouse strains in which involucrin or loricrin genes have been ablated [34].

The capacity for envelope formation appears to have arisen before the teleost-terrestrial tetrapod divergence as illustrated by the presence of horny teeth of agnathans [35], which exhibit detergent-resistant cell borders and contain abundant ε-(γ-glutamyl)lysine transglutaminase-mediated isopeptide bonds [36]. Tgm1-like transglutaminases in agnathans were not obtained from Genbank searches by the simple strategy employed, but further investigation in this direction appears warranted. Similarly, characterizing Tgm1-like genes in mudskipper and lungfish, better adapted to temporary existence outside an aqueous environment and closer to the mammalian lineage, would provide a useful contrast, especially since the latter species appears not to form horny features in its epidermis [37]. The finding of Tgm1-like genomic sequences encoding a cysteine cluster near the amino termini is striking, although the protein expression levels, possible alternate splicing and tissue distributions remain to be examined. Notably, the only sequences in some teleosts that are classified as Tgm1-like do not display the cysteine cluster near the amino terminus. Such species include *Lepisosteus oculatus* (spotted gar) and *Latimeria chalumnae* (coelacanth), the former from a more ancient lineage than teleosts and the latter considered more closely related to tetrapods than ray finned fishes [38]. Some teleosts have both types of Tgm1-like sequences, including *Scleropages formosus* (Table 1), likely reflecting divergence in sequence over time with potential adoption of new function. Such adoption could be enhanced by expression widely among tissues, as seen for medaka [7], unlike mammalian Tgm1 expressed primarily in the stratified squamous epithelia [39].

## Supporting information

S1 Fig Lack of envelopes in untransfected cells. In parallel with cultures transfected with lull length coding regions for Tgm1A or Tgm1B, untransfected HEK293 cultures were treated overnight with ionophore and then with SDS plus DTT. After shearing the DNA by passage through a 22 gauge needle, the samples were examined by phase contrast microscopy. Envelopes were not detected, since the cells completely dissolved. Control OmL cells treated with SDS and DTT without prior ionophore exposure also dissolved and lacked envelope structures when examined microscopically.
(PDF)

S2 Fig Translgutaminases in *O*. *niloticus*. The Genbank database of identified *O*. *niloticus* sequences was searched with BLASTP for the active site motif “GQCWVF”. Sequences are identified in the left margin by number: 1, 2, 3, 5, 13 (XIIIa) and NA (type not annotated); L, like; lower case letters arbitrarily distinguish those of the same type. The cysteine clusters near the amino termini of Tgm1-like (1La and 1Lb) sequences appear near residue 60 of the consensus, and the active site region used for searching is centered at residue 410. The sequences identified as transglutaminases were aligned using the MUSCLE multiple alignment tool through the software program Geneious 10.1 (https://www.geneious.com) for image export. The sequence with an inactivating cysteine to serine substitution in the active site (Genbank accession XP_005461247.1) was not included. Note that 5Lb and 5Lc differ by the former having a single extra amino acid, and thus they may not be distinct. Black highlighting indicates similar residues, dark gray indicates at least 80% but <100% similarity, light gray indicates 60–80% similarity, and white indicates <60% similarity.
(TIF)

S3 Fig Alignment of teleost Tgm1-related sequences. Genbank was searched using BLASTP for the active site motif “GQCWVF” with the “Organism” set for each of the 67 phylogenetic orders for bony fish given by Betancur et al (2014) [40]. From each of the 12 orders having Tgm1-like entries identified with cysteine clusters, an example was selected for display in Table 1 and aligned using MUSCLE multiple alignment tool through the software program Geneious 10.1 (https://www.geneious.com) for image export. For this purpose, only the C-terminal half is shown of the sequence (*I*.*punctatusB*) that appeared to be a fusion of two Tgm1-like sequences; the N-terminal half did not exhibit a cysteine cluster. A consensus sequence (requiring ≥85% identity) is provided for orientation. Exon-intron boundaries for the human sequence are indicated by vertical lines below the consensus. The cysteine cluster region shown in Table 1 is centered at residue ≈90 in the consensus, and the active site region used for searching is centered at residue 470. Accession numbers for the sequences are given in Table 1. A comparison is shown to the human, marsupial, amphibian and reptile sequences also included in the analysis of Table 1. Black highlighting indicates similar residues, dark gray indicates at least 80% but <100% similarity, light gray indicates 60–80% similarity, and white indicates <60% similarity.
(TIF)

S4 Fig Immunoblotting of tissue extracts. Freshly dissected lip (L), buccal (B) and opercular (O) tissues were heated in the presence of 2% sodium dodecyl sulfate and dithiothreitol and blotted as described in Methods using monoclonal antibody to either Tgm1A (upper panel) or Tgm1B (lower panel).
(PDF)

S1 Table Primer and probe sequences for gene expression assays. (PDF)

S2 Table Primer sequences for full length cDNA cloning. (PDF)

S3 Table Primer sequences for cDNA constructs to analyze amino terminal features. (PDF)

## References

[1] Makarova KS, Aravind L, Koonin EV (1999) A superfamily of archaeal, bacterial, and eukaryotic proteins homologous to animal transglutaminases. Protein Sci 8: 1714–1719. 10.1110/ps.8.8.1714 10452618 PMC2144420

[2] Korsgren C, Lawler J, Lambert S, Speicher D, Cohen CM (1990) Complete amino acid sequence and homologies of human erythrocyte membrane protein band 4.2. Proc Natl Acad Sci USA 87: 613–617. 2300550 10.1073/pnas.87.2.613 PMC53315

[3] Iismaa SE, Mearns BM, Lorand L, Graham RM (2009) Transglutaminases and disease: lessons from genetically engineered mouse models and inherited disorders. Physiol Rev 89: 991–1023. 10.1152/physrev.00044.2008 19584319

[4] Eckert RL, Kaartinen MT, Nurminskaya M, Belkin AM, Colak G, Johnson GV, et al (2014) Transglutaminase regulation of cell function. Physiol Rev 94: 383–417. 10.1152/physrev.00019.2013 24692352 PMC4044299

[5] Muszbek L, Bereczky Z, Bagoly Z, Komáromi I, Katona É (2011) Factor XIII: a coagulation factor with multiple plasmatic and cellular functions. Physiol Rev 91: 931–972. 10.1152/physrev.00016.2010 21742792

[6] Deasey S, Grichenko O, Du S, Nurminskaya M (2012) Characterization of the transglutaminase gene family in zebrafish and in vivo analysis of transglutaminase-dependent bone mineralization. Amino Acids 42: 1065–1075. 10.1007/s00726-011-1021-0 21809079 PMC3266987

[7] Kikuta A, Furukawa E, Ogawa R, Suganuma N, Saitoh M, Nishimaki T, et al (2015) Biochemical characterization of medaka (Oryzias latipes) transglutaminases, OlTGK1 and OlTGK2, as orthologues of human keratinocyte-type transglutaminase. PLoS One 10(12): e0144194 10.1371/journal.pone.0144194 26713442 PMC4694659

[8] Schmuth M, Gruber R, Elias PM, Williams ML (2007) Ichthyosis update: towards a function-driven model of pathogenesis of the disorders of cornification and the role of corneocyte proteins in these disorders. Adv Dermatol 23: 231–256. 10.1016/j.yadr.2007.07.011 18159904 PMC2603607

[9] Herman ML, Farasat S, Steinbach PJ, Wei MH, Toure O, Fleckman P, et al (2009) Transglutaminase-1 gene mutations in autosomal recessive congenital ichthyosis: summary of mutations (including 23 novel) and modeling of TGase-1. Human Mutation 30: 537–547. 10.1002/humu.20952 19241467 PMC3243309

[10] Chakravarty R, Rice RH (1989) Acylation of keratinocyte transglutaminase by palmitic and myristic acids in the membrane anchorage region. J Biol Chem 264: 625–629. 2462562

[11] Rice RH, Mehrpouyan M, O'Callahan W, Parenteau NL, Rubin AL (1992) Keratinocyte translglutaminase: Differentiation marker and member of an extended family. Epith Cell Biol 1: 128–137. 1364041

[12] Phillips MA, Qin Q, Mehrpouyan M, Rice RH (1993) Keratinocyte transglutaminase membrane anchorage: Analysis of site-directed mutants. Biochemistry 32: 11057–11063. 8105889 10.1021/bi00092a015

[13] Sun T-T, Green H (1976) Differentiation of the epidermal keratinocyte in cell culture: Formation of the cornified envelope. Cell 9: 511–521. 1009573 10.1016/0092-8674(76)90033-7

[14] Rice RH, Green H (1979) Presence in human epidermal cells of a soluble protein precursor of the cross-linked envelope: Activation of the cross-linking by calcium ions. Cell 18: 681–694. 42494 10.1016/0092-8674(79)90123-5

[15] Rice RH (1994) Assays for involucrin, transglutaminase and ionophore-inducible envelopes In: Leigh I. M. W FM, Lane B., editor. Keratinocyte Methods. UK: Cambridge University Press pp. 157–165.

[16] Gardell AM, Qin Q, Rice RH, Li J, Kültz D (2014) Derivation and osmotolerance characterization of three immortalized tilapia (*Oreochromis mossambicus*) cell lines. PLoS One 9(5): e95919 10.1371/journal.pone.0095919 24797371 PMC4010420

[17] Wong ML, Medrano JF (2005) Real-time PCR for mRNA quantitation. BioTechniques 39: 75–85. 16060372 10.2144/05391RV01

[18] Werner M, von Wasielewski R, Komminoth P (1996) Antigen retrieval, signal amplification and intensification in immunohistochemistry. Histochem Cell Biol 105: 253–260. 9072182 10.1007/BF01463928

[19] Werner M, Chott A, Fabiano A, Battifora H (2000) Effect of formalin tissue fixation and processing on immunohistochemistry. Am J Surg Pathol 24: 1016–1019. 10895825 10.1097/00000478-200007000-00014

[20] Carson F, Hladik Cappellano C (2015) Histotechnology, A Self-Instructional Text: American Society for Clinical Pathology.

[21] Lien S, Koop BF, Sandve SR, Miller JR, Kent MP, Nome T, et al (2016) The Atlantic salmon genome provides insights into rediploidization. Nature 533: 200–205. 10.1038/nature17164 27088604 PMC8127823

[22] Macqueen DJ, Johnston IA (2014) A well-constrained estimate for the timing of the salmonid whole genome duplication reveals major decoupling from species diversification. Proc Biol Sci 281: 20132881 10.1098/rspb.2013.2881 24452024 PMC3906940

[23] Green H (1977) Terminal differentiation of human epidermal cells. Cell 11: 405–415. 302145 10.1016/0092-8674(77)90058-7

[24] Rice RH, Green H (1978) Relationship of protein synthesis and transglutaminase activity to formation of the cross-linked envelope during terminal differentiation of the cultured human epidermal keratinocyte. J Cell Biol 76: 705–711. 24643 10.1083/jcb.76.3.705 PMC2110014

[25] Phillips MA, Stewart BE, Rice RH (1992) Genomic structure of keratinocyte transglutaminase. Recruitment of new exon for modified function. J Biol Chem 267: 2282–2286. 1346394

[26] Ono RD (1980) Fine structure and distribution of epidermal projections associated with taste buds on the oral papillae in some loricariid catfishes (SiIuroidei: Loricariidae). J Morphol 164: 139–159. 30170476 10.1002/jmor.1051640204

[27] Roberts TR (1982) Unculi (horny projections arising from single cells), an adaptive feature of the epidermis of Ostariophysan fishes. Zoologica Scripta 11: 55–76.

[28] Tripathi P, Mittal AK (2010) Essence of keratin in lips and associated structures of a freshwater fish Puntius sophore in relation to its feeding ecology: Histochemistry and scanning electron microscope investigation. Tissue Cell 42: 223–233. 10.1016/j.tice.2010.04.005 20684836

[29] Wiley ML, Collette BB (1970) Breeding tubercles and contact organs in fishes: their occurrence, structure and significance. Am Mus Nat Hist 143: 143–216.

[30] Fischer B, Metzger M, Richardson R, Knyphausen, Ramezani T, Franzen R, et al (2014) p53 and TAp63 promote keratinocyte proliferation and differentiation in breeding tubercles of the zebrafish. PLoS Genetics 10(1): e1004048 10.1371/journal.pgen.1004048 24415949 PMC3886889

[31] Phillips MA, Stewart BE, Qin Q, Chakravarty R, Floyd EE, Jetten AM, et al (1990) Primary structure of keratinocyte transglutaminase. Proc Natl Acad Sci USA 87: 9333–9337. 1979171 10.1073/pnas.87.23.9333 PMC55159

[32] Mittal AK, Whitear M (1979) Keratinization of fish skin with special reference to the catfish Bagarius bagarius. Cell Tissue Res 202: 213–230. 519704 10.1007/BF00232236

[33] Eckhart L, Lippens S, Tschachler E, Declercq (2013) Cell death by cornification. Biochim Biophys Acta 1833: 3471–3480. 10.1016/j.bbamcr.2013.06.010 23792051

[34] Rice RH, Durbin-Johnson BP, Ishitsuka YI, Salemi M, Phinney BS, Rocke DM, et al (2016) Proteomic analysis of loricrin knockout mouse epidermis. J Proteome Res 15: 2560–2566. 10.1021/acs.jproteome.6b00108 27418529

[35] Dawson JA (1963) The oral cavity, the ‘jaws’ and the horny teeth of *Myxine glutinosa* In: Brodal A, Fänge R, editors. The Biology of Myxine. Oslo: Scandinavian University Books pp. 231–255.

[36] Rice RH, Wong VJ, Pinkerton KE (1994) Ultrastructural visualization of cross-linked protein features in epidermal appendages. J Cell Sci 107: 1985–1992. 7983163 10.1242/jcs.107.7.1985

[37] Alibardi L, Joss JM (2003) Keratinization of the epidermis of the Australian lungfish Neoceratodus forsteri (Dipnoi). J Morphol 256: 13–22. 10.1002/jmor.10073 12616571

[38] Amemiya CT, Alföldi J, Lee AP, Fan S, Philippe H, MacCallum I, et al (2013) The African coelacanth genome provides insights into tetrapod evolution. Nature 18: 311–316. 10.1038/nature12027 PMC3633110 23598338

[39] Parenteau NL, Pilato A, Rice RH (1986) Induction of keratinocyte type-I transglutaminase in epithelial cells of the rat. Differentiation 33: 130–141. 2436965 10.1111/j.1432-0436.1986.tb00418.x

[40] Betancur-R R, Wiley E, Bailly B, Miya M, Lecointre G, Ortí G (2014) Phylogenetic Classification of Bony Fishes Fish Base. Version 3 ed: DeepFin.org. 10.1186/s12862-017-0958-3 PMC5501477 28683774

