# Supplementary material for: Tgm1-like transglutaminases in tilapia (Oreochromis mossambicus)
Source: PLoS One. 2017 May 4;12(5):e0177016. doi: 10.1371/journal.pone.0177016 (PMC5417640; doi:10.1371/journal.pone.0177016)

S1 Figure. Lack of envelopes in untransfected HEK293FT cells. In parallel with cultures transfected with lull length coding regions for Tgm1A or Tgm1B, untransfected cultures were treated overnight with ionophore and then with SDS plus DTT. After shearing the DNA by passage through a 22 gauge needle, the samples were examined by phase contrast microscopy at the same magnification as transfected cultures. Envelopes were not detected, since the cells completely dissolved. Control OmL cells treated with SDS and DTT without prior ionophore exposure, which also dissolved, also lacked envelope structures when examined microscopically.

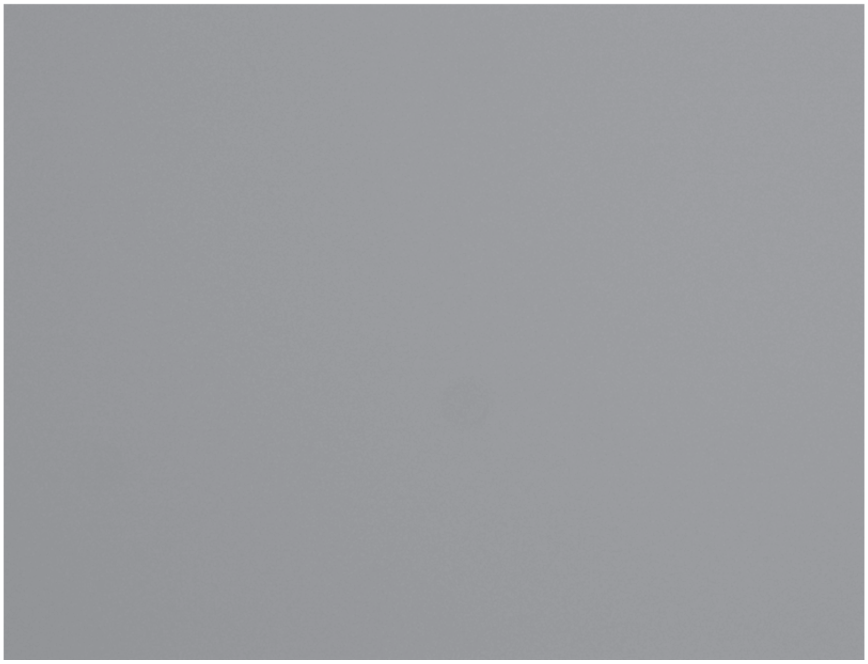

Supplement: S1 Fig — In parallel with cultures transfected with lull length coding regions for Tgm1A or Tgm1B, untransfected HEK293 cultures were treated overnight with ionophore and then with SDS plus DTT. After shearing the DNA by passage through a 22 gauge needle, the samples were examined by phase contrast microscopy. Envelopes were not detected, since the cells completely dissolved. Control OmL cells treated with SDS and DTT without prior ionophore exposure also dissolved and lacked envelope structures when examined microscopically. (PDF) [file pone.0177016.s001.pdf]
