# Supplementary figures and images for: Tgm1-like transglutaminases in tilapia (Oreochromis mossambicus)
Source: PLoS One. 2017 May 4;12(5):e0177016. doi: 10.1371/journal.pone.0177016 (PMC5417640; doi:10.1371/journal.pone.0177016)

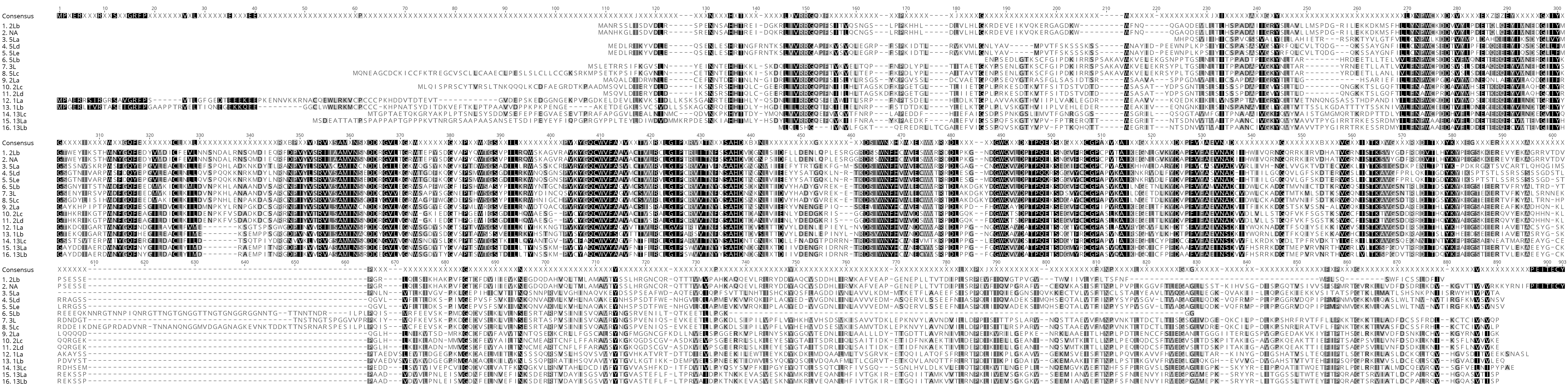

Supplement: S2 Fig — The Genbank database of identified O. niloticus sequences was searched with BLASTP for the active site motif “GQCWVF”. Sequences are identified in the left margin by number: 1, 2, 3, 5, 13 (XIIIa) and NA (type not annotated); L, like; lower case letters arbitrarily distinguish those of the same type. The cysteine clusters near the amino termini of Tgm1-like (1La and 1Lb) sequences appear near residue 60 of the consensus, and the active site region used for searching is centered at residue 410. The sequences identified as transglutaminases were aligned using the MUSCLE multiple alignment tool through the software program Geneious 10.1 (https://www.geneious.com) for image export. The sequence with an inactivating cysteine to serine substitution in the active site (Genbank accession XP_005461247.1) was not included. Note that 5Lb and 5Lc differ by the former having a single extra amino acid, and thus they may not be distinct. Black highlighting indicates similar residues, dark gray indicates at least 80% but <100% similarity, light gray indicates 60–80% similarity, and white indicates <60% similarity. (TIF) [file pone.0177016.s002.tif]

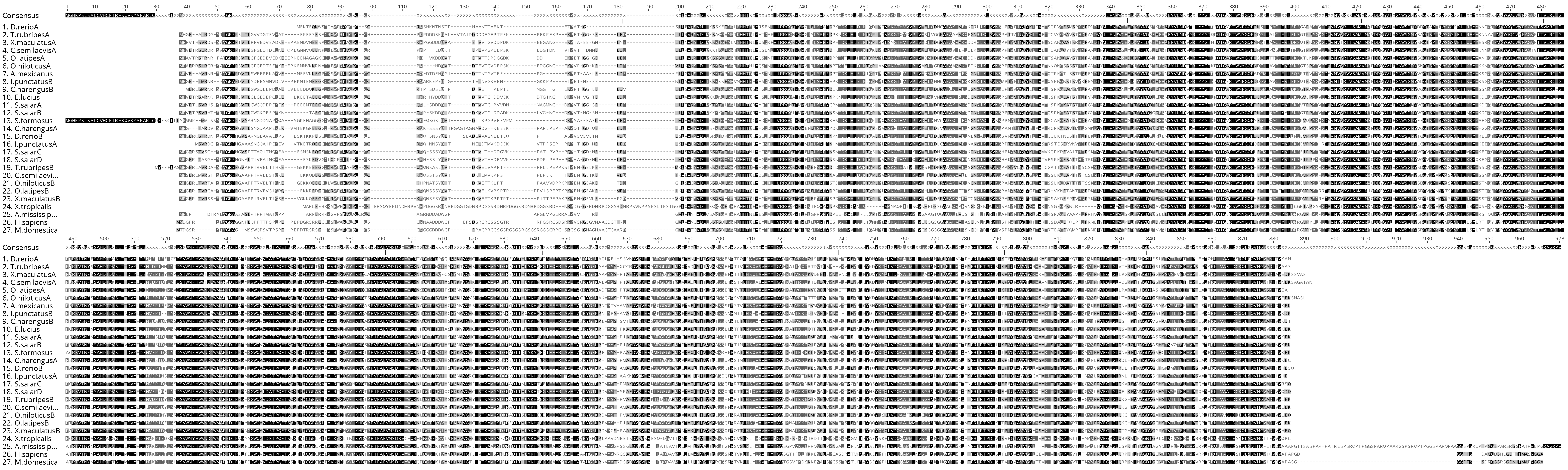

Supplement: S3 Fig — Genbank was searched using BLASTP for the active site motif “GQCWVF” with the “Organism” set for each of the 67 phylogenetic orders for bony fish given by Betancur et al (2014) [40]. From each of the 12 orders having Tgm1-like entries identified with cysteine clusters, an example was selected for display in Table 1 and aligned using MUSCLE multiple alignment tool through the software program Geneious 10.1 (https://www.geneious.com) for image export. For this purpose, only the C-terminal half is shown of the sequence (I.punctatusB) that appeared to be a fusion of two Tgm1-like sequences; the N-terminal half did not exhibit a cysteine cluster. A consensus sequence (requiring ≥85% identity) is provided for orientation. Exon-intron boundaries for the human sequence are indicated by vertical lines below the consensus. The cysteine cluster region shown in Table 1 is centered at residue ≈90 in the consensus, and the active site region used for searching is centered at residue 470. Accession numbers for the sequences are given in Table 1. A comparison is shown to the human, marsupial, amphibian and reptile sequences also included in the analysis of Table 1. Black highlighting indicates similar residues, dark gray indicates at least 80% but <100% similarity, light gray indicates 60–80% similarity, and white indicates <60% similarity. (TIF) [file pone.0177016.s003.tif]
