# Supplementary material for: Tgm1-like transglutaminases in tilapia (Oreochromis mossambicus)
Source: PLoS One. 2017 May 4;12(5):e0177016. doi: 10.1371/journal.pone.0177016 (PMC5417640; doi:10.1371/journal.pone.0177016)

S4 Figure. Immunoblotting of Tissue Extracts. Freshly dissected lip (L), buccal (B) and opercular (O) tissues were heated in the presence of 2% sodium dodecyl sulfate and dithiothreitol and blotted as described in Methods using monoclonal antibody to either Tgm1A (upper panel) or Tgm1B (lower panel).

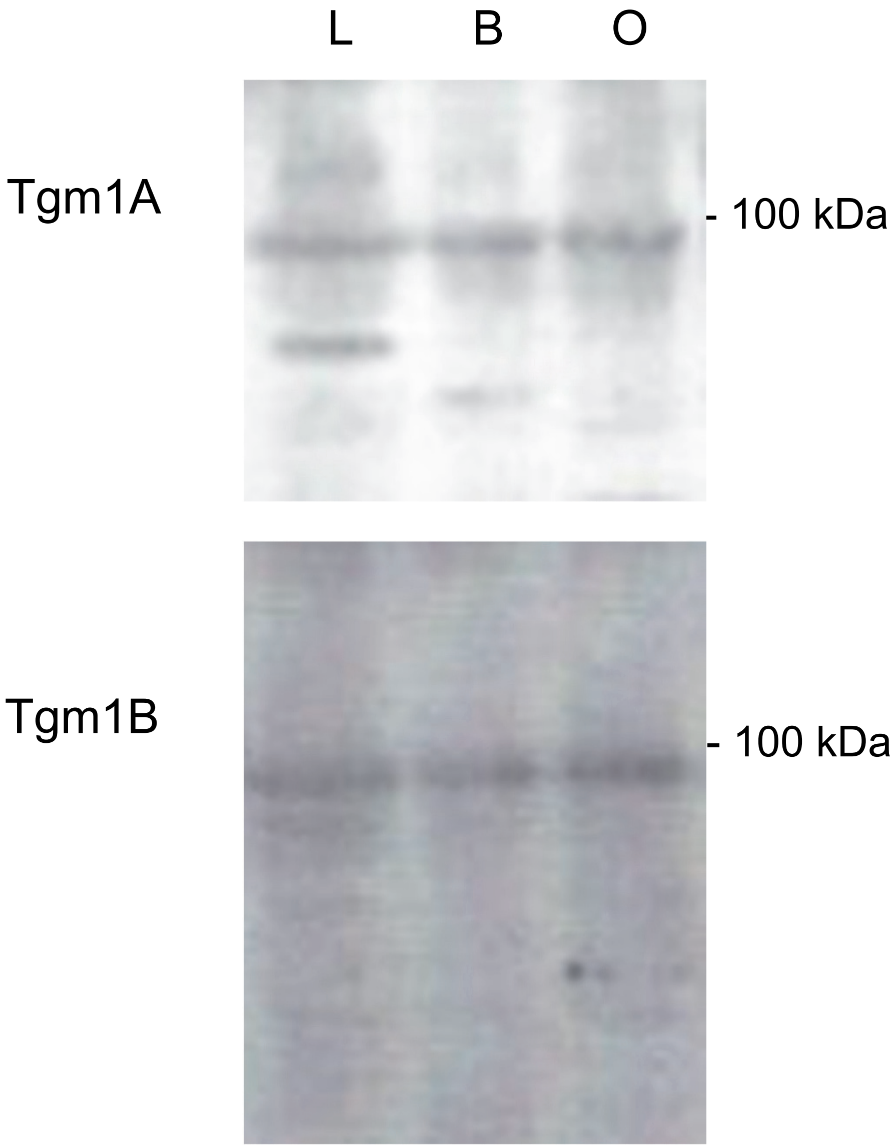

Supplement: S4 Fig — Freshly dissected lip (L), buccal (B) and opercular (O) tissues were heated in the presence of 2% sodium dodecyl sulfate and dithiothreitol and blotted as described in Methods using monoclonal antibody to either Tgm1A (upper panel) or Tgm1B (lower panel). (PDF) [file pone.0177016.s004.pdf]
