# Supplementary material for: Tgm1-like transglutaminases in tilapia (Oreochromis mossambicus)
Source: PLoS One. 2017 May 4;12(5):e0177016. doi: 10.1371/journal.pone.0177016 (PMC5417640; doi:10.1371/journal.pone.0177016)
Supplement: S1 Table — (PDF) [file pone.0177016.s005.pdf]

S1 Table. Primer and Probe Sequences for Gene Expression Assays[a].

Gene	Forward	Reverse	Reporter
Tgm1A	CCACCTCCGATGAGCTTCA	ATGAGTTCCCTTTGCCACTACTG	CCAGTGGACCTGTTTTAA
Tgm1B	CTCCAGCAAGAACATCGTCATG	CCCCTCGTTATCCAGGAACAC	CTTGGTGTGAAGAGGACAC
Talin1	GCATTTTGGATGTCACTGAGAACA	CGAGCCTGACGGACCAT	CTCACCAGCATCTCCC
FilA	TGCACCGTCACAGTCTCAATC	TCTGTTCGCCAATCTGGATAGTTG	ACCGGCACCTAAACCG
S2-40S	CTGCACCGCCACCCT	GTAAGTCTTGGAGATGGCGTCAAA	ACTTTGCCAAGGCCACC

[a]Custom designed Taqman Assays provided by Thermo Fisher Scientific (Applied Biosystems). All amplicons are in the range 61-76 bp.
