# Supplementary material for: Tgm1-like transglutaminases in tilapia (Oreochromis mossambicus)
Source: PLoS One. 2017 May 4;12(5):e0177016. doi: 10.1371/journal.pone.0177016 (PMC5417640; doi:10.1371/journal.pone.0177016)
Supplement: S2 Table — (PDF) [file pone.0177016.s006.pdf]

S2 Table. Primer Sequences for Full Length cDNA Cloning[a].

Gene	Forward	Reverse
Tgm1A	*AGACCCAAGCTGGCTAGC*ATGCCTGCAGAGAGGCG	*GTGGCGGCCGCTCGAG*TAGAGAGGCGTTGCTTTTCTCC
Tgm1B	*AGACCCAAGCTGGCTAGC*ATGCCAGGTGAACGGTTAAC	*GTGGCGGCCGCTCGAG*TTGTTCAAGAACAACAATGTCAGC
pcDNA3.1	CTCGAGCGGCCGCCAC	GCTAGCCAGCTTGGGTCTCCC

[a]The italicized portions of the Tgm1 primers were added to create overlaps with the cloning vector which are required for the Gibson assembly reaction. Accession numbers for Tgm1A and Tgm1B are XM_005478896.1 and XM_003456177.3, respectively.
