# Supplementary material for: Tgm1-like transglutaminases in tilapia (Oreochromis mossambicus)
Source: PLoS One. 2017 May 4;12(5):e0177016. doi: 10.1371/journal.pone.0177016 (PMC5417640; doi:10.1371/journal.pone.0177016)
Supplement: S3 Table — (PDF) [file pone.0177016.s007.pdf]

S3 Table. Primer sequences for cDNA Constructs to Analyze Amino Terminal Features

A. Delete Cysteine Cluster (CPCCC)

Gene	Forward	Reverse
Tgm1A	CCCAAGCATGATGATGTCAC	GACCTTACGCAGCCACTC
Tgm1B	AAGCACCCAAACGCCACC	CATCTTTCTGAGCCACCATAGAC

B. Mutagenize Cysteine Cluster (AIAAA) and Introduce MscI Site

Gene	Forward	Reverse
Tgm1A	CTGCCGCCCCCAAGCATGATGATGTCAC	CAATGGCCACCTTACGCAGCCACTCC
Tgm1B	TGCCGCCAAGCACCCAAACGCCACC	GCAATGGCCATCTTTCTGAGCCACCATAGAC

C. Remove N-Terminal Residues 3-94

Gene	Forward	Reverse
Tgm1A	GAAGTCCTTCTCAAAGTGAAGTCAATAG	AGGCATGCTAGCCAGCTT
Tgm1B	GAAACAGATGAAGGGAAGCTG	TGGCATGCTAGCCAGCTT
